# Strategies for Identifying Core Components of Programs: an Exploratory Descriptive Component Case Study of a Teen Pregnancy Prevention Program

**DOI:** 10.1007/s11121-026-01881-8

**Published:** 2026-02-10

**Authors:** Russell Cole, Jean Knab, Emily Forrester

**Affiliations:** Mathematica Inc., 600 Alexander Park, Suite 100, Princeton, NJ USA

**Keywords:** Program components, Measurement, Outcomes, Implementation, Prospective study, Retrospective study, Analysis, Case study

## Abstract

Studies of program components (i.e., the ingredients that make up programs) have risen from obscurity to join mainstream program evaluation approaches over the last two decades. Researchers and policymakers are interested in leveraging information about the effectiveness of program components to better address the needs of programs’ intended populations and reduce disparities in outcomes. Identifying which components are responsible for improving outcomes can inform evaluation design, measurement, and the state of the evidence as well as program development, adaptation, fidelity, and scale-up. This paper summarizes strategies for conducting components research, and anchors those emerging best practices in a components case study of a teen pregnancy prevention program. It offers applied guidance on ways to define and operationalize components, and best practices in measurement and analysis that have emerged from this work. This paper will help guide the prevention field toward conducting more and better research that yields consensus about which components are most important for program effectiveness.

**Supplementary Information:**

The online version contains supplementary material available at 10.1007/s11121-026-01881-8.

## Introduction

The evidence base for prevention programs has grown substantially over the past several decades. Practitioners have at their fingertips multiple online registries and systematic reviews (for example, the Office of Juvenile Justice and Delinquency Prevention’s Model Programs Guide, the Substance Abuse and Mental Health Services Administration’s Evidence-based Practice Center, the California Evidence-Based Clearinghouse for Child Welfare, the Trust for America’s Health report on community-based prevention programs) that provide information about evidence-based curricula, programs, and practices (hereafter, programs) and the evidence that supports their use.

Less understood is why one program is effective and another is not. What is the “secret sauce” that makes some programs effective? Across prevention science, there is growing recognition that identifying and articulating the core components of evidence-based programs (EBPs) is critical to advancing replication, adaptation, and equitable scale-up (Blase & Fixsen, 2013). Core components—defined as the essential elements responsible for a program’s effectiveness—can be conceptualized as the building blocks of an effective intervention or practice (Forrester & Cole, 2023b; Lemire et al., 2025).

Knowing which components of a program are core (and thus, must be implemented to achieve intended outcomes) can help program developers create effective curricula, inform whether and how to permit program adaptations, or help practitioners enhance existing programs by monitoring the implementation of the core components as part of continuous quality improvement. In addition, in some instances, core components can be implemented as standalone services or supplements to programs to address programmatic needs and service gaps (Asheer & Aharpour, 2025). Effectively implementing some components of a program might be a way to address populations’ disparities in outcomes and reduce service inequities when the full program is too expensive or infeasible to implement in a given setting (Butler, 2020; Castillo-Sumi, 2020; Wilson et al., 2020).

There have been several instructional publications in recent years to help guide the emerging body of components research. Ferber et al. (2019) described different ways of using core components to inform program development and implementation and offered guidance on ways to identify them in programs. Dymnicki et al. (2020) provide suggestions for ways to identify and test components as predictors of outcomes. Cole and Choi (2020) outlined three different analytic approaches to identify potentially promising core components of programs. More recently, Lemire et al. (2025) presented a landscape of approaches to identify effective components. A key commonality in this work is that all authors called for better documentation of components and called for more research that shows credible connections between components and outcomes (in other words, determining which components are core).

Our team conducted a prospective component study to document the components of a teen pregnancy prevention (TPP) program and link those components to outcomes. In doing so, we learned that defining components, measuring variation in component experiences, and credibly linking those components to outcomes is complex and multi-faceted in practice, and that additional guidance is needed to address these gaps. In this manuscript, we use the Components Study of REAL Essentials Advance (Components Study of REA) as a case study to illustrate the practical considerations for component identification, measurement, and analysis, and offer conclusions about best practices for future components research.

### Approach and Goals for This Paper

This paper aims to present practical considerations for researchers designing and executing an exploratory component study of an individual program, anchored in a real-world example. In the remainder of this paper, we first briefly discuss current approaches commonly used to examine components of interventions. Then, we provide a brief overview of the Components Study of REA, before presenting practical considerations when designing and executing a component study through real examples from this case study. We present approaches and guidance for operationalizing and measuring components, and methodological approaches to linking component experiences to outcomes. We conclude with recommendations for the field.

Table 1 presents definitions used in the remainder of the article.

**Table 1:** Definitions of component terms

Term	Definition
Component	The elements and activities that constitute a program
Core component	The subset of components responsible for a program’s effectiveness
Structural element	Well defined, mutually exclusive, and exhaustive units that, when combined, can be conceptualized as the intended experience of the program
Component type	A frame to conceptualize programs in terms of content, delivery mechanism, format, staffing, dosage, environment, and intended population characteristic
Component category	A grouping of substantively similar, fine-grained components into a broader, more easily understood bucket within their broader categorization as individual components of the same component type

### Recent Approaches to Understanding Program Components

Recent guidance on identifying components is largely focused on looking across multiple programs in a field at once (see Lemire et al., 2025 for a review). Our article focuses on best practices for identifying components of a single program. However, we considered and drew on some of the approaches mentioned in the literature below to inform our approach.

The distillation and matching method (DMM, Chorpita et al., 2005) is a common approach for identifying common components of multiple programs—it was initially used to identify components of mental health programs. In this approach, interventions are first coded into their core components (the distillation process), and then programs with similar sets of components are grouped together (the matching process). See Oddo et al., 2025 for a recent example of this approach used to identify common components of prevention programs for youth social, emotional, and behavioral problems, Lawson et al., 2019 for an example of this approach for social-emotional learning programs, and Forrester et al., 2024 as an example of this approach in the TPP context.

Another approach for identifying common components of multiple programs, particularly those components that appear to be driving effectiveness, is meta-regression (Higgins et al., 2021). In a component meta-regression, program effectiveness findings are regressed on predictor variables that represent the components of the programs being examined. The approach estimates the contribution of each component on the impact findings and therefore can identify those core components of programs that appear to be driving larger (or smaller) average effects. See Walsh et al. (2023) for an example of a meta-regression focused on components of early literacy interventions, Wilson et al. (2020) for an examination of components of programs intended to reduce externalizing behavior problems, Cole et al. (2024) for an example of content components of TPP programs, and Stanczyk et al. (2021) for an exploration into the promising components of employment programs.

Both DMM and meta-regression require programs to be disaggregated into their components as a first step. This disaggregation can be conducted as a top-down approach, where an existing taxonomy of components is used as the reference against which all programs are compared (and programs can be coded as including or not including a given component in the taxonomy), or it can be a bottom-up approach where the components naturally emerge from an examination of program documentation.

In a top-down approach, an existing framework or taxonomy of components is used to create a structured classification of the components of a given set of programs. With a taxonomy as a guide, it is feasible to look across programs and code whether or not a given program includes certain components (for example, a type of content, activity, or format). Abraham and Michie (2008) and Kok et al. (2015) have a broad taxonomy that can be applied to code interventions focused on behavior change. Scher and Martinez (2022) created a taxonomy to code components of education interventions, and Visscher et al. (2018) created a taxonomy for programs intended to address families facing multiple problems. Forrester and Cole (2023b) created a taxonomy for TPP programs—we discuss this taxonomy in more detail below in its use in the Components Study of REA.

On the other hand, coding the components of programs can take a more bottom-up approach. EBP intervention mapping (IM) is an example of this (Walker et al., 2022). EBP IM is an extension of general IM, a step-by-step process for developing interventions (Bartholomew Eldredge et al., 2016; Fernandez et al., 2019). EBP IM is used to reverse engineer EBP programs into their individual components. The approach for identifying components in EBP IM is a generative, bottom-up approach based on identifying the change methods, design features, and implementation strategies of each program that are likely to affect outcomes, rather than using a list of components found in a taxonomy. Guidance from implementation science can inform which components might be most useful (Proctor et al., 2013). See Szeszulski et al. (2022) as an application of EBP IM to identify core components of colorectal cancer screening interventions.

Beyond coding components of programs, researchers also require guidance on analytic approaches to link components to outcomes in studies of single programs (since DMM and meta-regression are used for analyses of multiple programs). One conceptual idea is to see components as part of the integrated model of program implementation (Berkel 2011), and to empirically estimate their relationships with outcomes (potentially taking into account variation in fidelity and quality or dosage as ways to understand the roles of components as predictors). Ferber et al. (2019), Dymnicki et al. (2020), Cole and Choi (2020), and Cole and Murphy (2016) offer guidance on analyses that build on this framework.

Other approaches to study components focus on prospective component studies with manipulation of components to condition, where researchers have a-priori hypotheses about which components are the most important and wish to rigorously test those components. See Gryczynski et al. (2021) and DiClemente et al. (2014) as examples of randomized experiments that investigated individual components of TPP programs. More complex approaches can also be used when multiple components are added in an adaptive and interactive way to achieve a maximally effective program. Approaches such as the Multiphase Optimization Strategy (known as MOST) (Collins et al., 2011, 2014) and the Sequential Multiple Assignment Randomized Trial (known as SMART) (Almirall et al., 2012; Lei et al., 2012) can operationalize these more complex evaluations requiring manipulation of components. Alternatively, in this article, we focus on considerations when examining the components of a single program in an exploratory manner. Approaches to rigorously test promising components might be particularly useful or informative as a logical follow-on to the types of exploratory component studies described here and presented in the remainder of this article.

The case study examined in this paper focused on the measurement of the components of a single program using a top-down taxonomy, and an exploratory analysis of the relationship between components experienced and outcomes to identify those that had the strongest empirical linkages. Although the general approaches used in this study for measuring components were informed by the approaches described in the literature above, we found that in practice, more specific and concrete guidance was needed. We developed such guidance while conducting this case study, and share our learnings in this article to address this gap for future researchers conducting comparable explorations of the component drivers of outcomes of individual programs.

## The Motivating Study: Components Study of REA

The Components Study of REA was a descriptive implementation and outcome study funded by the Office of Population Affairs (OPA) in 2020. The goal of the study was to identify individual components of the REAL Essentials Advance (REA) curriculum that promote positive health behaviors and other outcomes. REA covers much of the content covered by other TPP programs. Determining which components in REA are associated with youth outcomes could help inform OPA and curriculum developers about what curriculum adaptations to (dis)allow among the broader set of TPP curricula. It would also inform the field more broadly about important components to include in future curricula and to highlight the importance of implementing these core components well in existing curricula.

The REA curriculum is a healthy relationship curriculum designed for high school age youth (Center for Relationship Education, n.d.), and includes 87 lessons of varying lesson lengths spanning multiple topics (Cole et al., 2025b). The curriculum is intentionally flexible so that organizations can select the subset of lessons that best meet the needs of the youth receiving it. Organizations implementing the program, in conjunction with the developer, create a customized scope and sequence (S&S), which is a collection and ordering of a subset of the 87 REA lessons selected to address the needs of the intended population (in terms of desired content) and within available implementation constraints (e.g., duration of programming and length of each implementation session). Because organizations can implement REA differently (through unique S&Ss that contain varied collections of lessons), youth receiving REA are exposed to different components: content, activities, duration, staff characteristics, contexts, and so on. We hypothesized that the variation in component experiences would yield variation in outcomes, allowing us to identify which components are core for which outcomes. For example, organizations that offer lessons on social media and bullying (or more lessons on those topics) might show larger improvements in outcomes related to that type of content than organizations that do not (or offer less content).

We recruited schools to implement REA in spring and fall semesters of 2022 and asked them to prioritize among topics of interest covered by the curriculum. Then the curriculum developer or its lead facilitators assembled a S&S based on the school’s priorities and time constraints. We measured outcomes using surveys administered before the program, immediately after the program, and six months after the program ended. The surveys included a wide array of outcomes expected to be affected by the various types of content the curriculum covered. In the analysis, we linked component experiences to outcomes and estimated how variation in component experience was predictive of outcomes (see subsequent sections below for information on how we operationalized components and outcomes in this study).

The Components Study of REA generated a large number of useful findings about components of TPP programs. Anastasio et al. (2025) highlighted different types of content that were strongly associated with outcome improvement, including, but not limited to, substance use and emotional health content as potential drivers of several outcomes. This report also highlighted that interactive activities and longer dosage duration were components that were strongly predictive of multiple outcomes. Walzer et al. (2025) built on these findings to show that interactive activity components were the ones that drove the highest levels of youth engagement, and that it was through engagement as a mediating variable that improvements in outcomes were being observed. Asheer and Blesson (2025) focused on qualitative data to help identify the features of facilitators, and the methods by which components are delivered played a role in outcome improvement. Finally, Knab et al. (2025) provide an overview of the variety of components that were examined in this study, as observed across all of the participating schools. This summary only begins to scratch the surface of the many results that emerged out of the Components Study of REA—there are many more, and far more detailed findings found in each of these individual reports.

### Case Study

The Components Study of REA is a useful case study to illustrate a collection of complexities in a real-world prospective component study. The flexible REA curriculum was uniquely suited to facilitate a components study, and the study design created a wide variety of component experiences. In turn, the study yielded a number of emerging best practices around documenting program components. The approaches described here can be readily transferred to document components of other prevention programs. This paper also offers insight into measurement of appropriate outcomes that will be sensitive to change, offering guidance for future prospective component studies of prevention programs. The design, measurement approaches, and analytic approaches we describe were informed and vetted by multiple expert panels. This paper provides a unique opportunity for readers to learn these best practices from the lessons that we learned when designing and conducting this study.

### Operationalizing Program Components

**Fig. 1:**
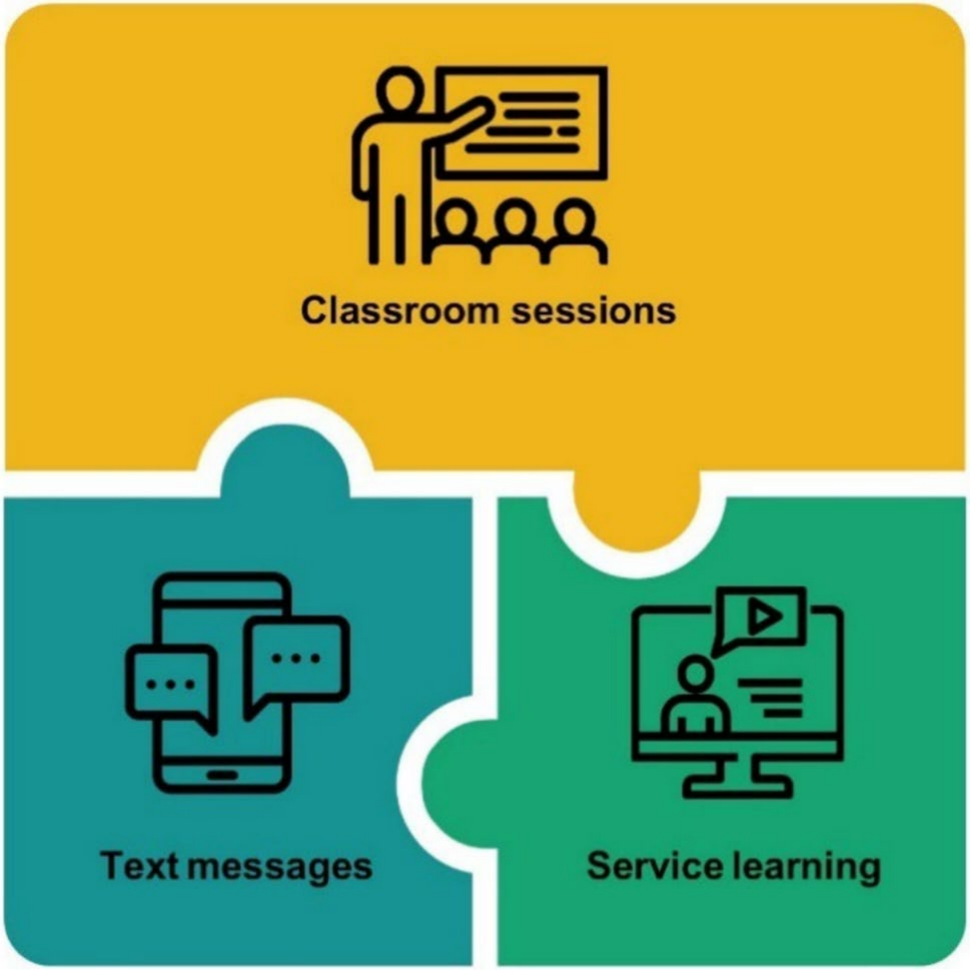
Structural elements of an illustrative TPP program

During the design phase of the Components Study of REA, we explored two options for operationalizing program components of the REA curriculum. One way to conceptualize components is by breaking up a program into its smaller structural elements (Cole & Murphy, 2016) in a bottom-up process. Structural elements are well defined, mutually exclusive, and exhaustive units that, when combined, are the intended experience of the program. Structural elements are those that could, in theory, operate as stand-alone portions of programs. For example, a program that includes a curriculum, a service-learning project, and text messaging boosters has three distinct structural elements. They can be thought of as independent pieces of a puzzle that, when combined, represent all the experiences that constitute a program (Fig. 1). Structural elements could also be measured at finer levels, for instance, breaking up a multi-lesson curriculum into specific lessons. A key feature of structural elements is that each has a clear boundary, and they are each easily labeled and understood experiences that offer immediate insight into the smaller elements that comprise the whole program.

A top-down approach offers an alternative way to abstract program components by using commonly understood features from a broader taxonomy (NASEM, 2020; Forrester & Cole, 2023a). For example, we can separate common features of prevention programs into seven component types: (1) content, (2) delivery mechanisms, (3) formats, (4) staffing, (5) dosages, (6) environments, and (7) intended population characteristics. Extending the example from above, across the illustrative TPP program’s classroom sessions, text messages, and service-learning structural elements, there is content delivered in a certain format by a staff member (with certain characteristics) for a certain dosage (amount of time). Within each of those component types, we can articulate individual components. An individual format component, for example, is online (synchronous).

**Fig. 2:**
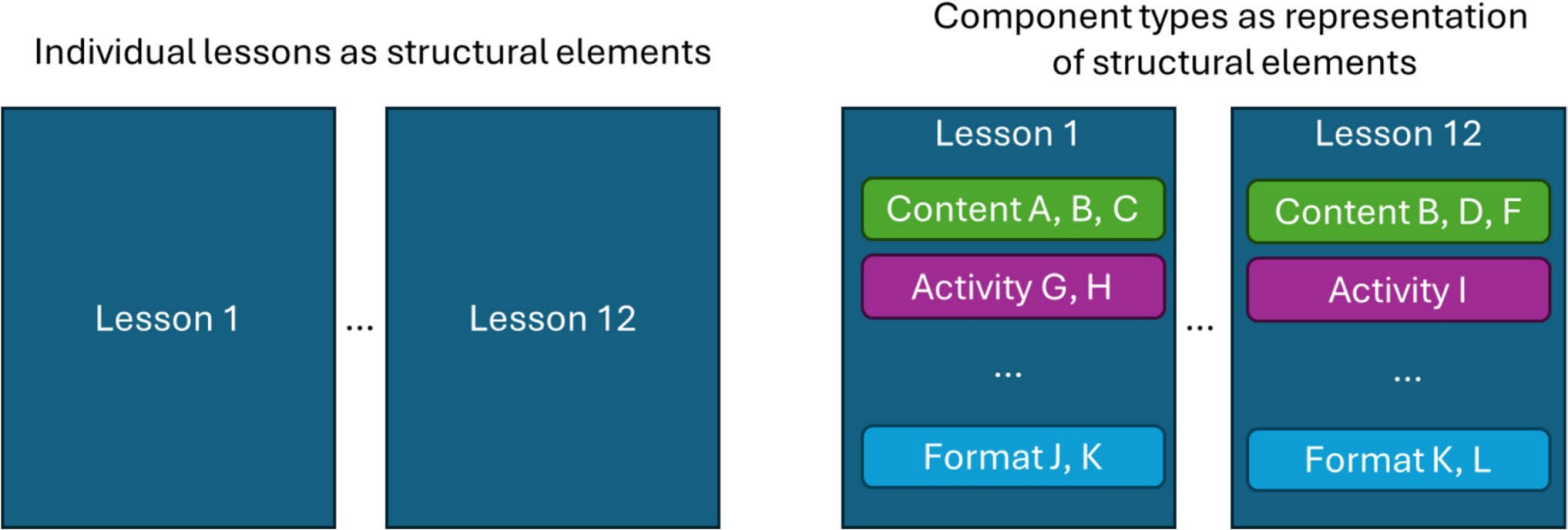
Structural elements versus component types

The Components Study of REA incorporated aspects of both structural elements and component types to operationalize the REA program components. Individual lessons of the REA curriculum made up the unique structural elements that youth participants experienced. Youth varied in terms of the combinations of lessons that they experienced, resulting from variation in the S&S offered at a given school (and their attendance of those lessons). However, most audiences (except those extremely familiar with the REA program) would not be interested in whether youth who received an S&S containing lessons A, B, and C had outcomes better than youth who received a different S&S containing lessons A, E, and F, for example. That is, focusing on lessons as structural elements of REA would not yield a useful/interpretable story about components (whereas in the previous example of a three structural element program containing a curriculum, service-learning project, and text messaging boosters might be more useful/informative to a broader audience, given a more universal understanding of what those elements are). Therefore, the individual components that make up the content, activities, and dosage component types offered across the 87 lessons of the REA curriculum were used as the components of interest for the study. This approach offered an opportunity to examine fine-grained components and present findings that would potentially be useful and applicable outside of the context of the REA program (Fig. 2).

### Measuring Program Components

To measure the individual components within the Components Study of REA, we used a taxonomy of components of TPP programs: the TPP program components checklist. The checklist is a disaggregation tool that can be used to consistently and reliably code the individual program component types that make up a whole program. Below we describe (1) the considerations and approaches taken to develop and refine the TPP program component checklist and (2) how we used the component checklist to reliably measure components’ experiences in the Component Study of REA.

#### The Development and Refinement of the TPP Component Checklist

Before the Components Study of REA began, we developed a standardized checklist for program developers and distributors to use to classify their TPP programs into components (Forrester & Cole, 2023a). This list was initially informed by the assembled characteristics of optimal adolescent health programs identified through the NASEM 2020 review of meta-analyses and systematic reviews (NASEM, 2020). We worked with practitioners, developers, and researchers in the field to refine the NASEM list of program characteristics to focus on the topics and approaches most common to TPP programs, specifically, and reviewed several TPP curricula to identify and add components not in the original list. We then categorized program components into the seven broad types described earlier.

To vet the utility and appropriateness of the checklist, we convened a panel and held multiple meetings. Panelists included program developers, practitioners, researchers, and youth participants of TPP programs. We piloted the checklist internally, and we asked panelists to test the checklist by coding the components of an evidence-based TPP program they were most familiar with. We worked with the panelists over the course of two meetings to produce successive iterations of the checklist. Changes included refining component definitions, adding components that were missing from the list, and improving the instructions that accompany the checklist. These revisions addressed experts’ concerns about the potential for the checklist to be a reliable measurement tool, so that two (or more) people could use the tool to code components that make up a program, using the same documentation, and arrive at the same collection of components. After we revised the checklist in response to expert feedback, we prepared it for dissemination.

The ultimate checklist includes 166 individual components across the seven broad types of components. For example, the checklist includes 69 individual content components (such as condoms, maternal health, and sexual orientation) and 31 individual delivery mechanism components (such as demonstration, lecture, and role-play). The checklist is available as online appendix A.1.

Thus, the checklist can be used to code the fine-grained components of a program as a whole, or in the case of REA, each individual lesson, in terms of each component type (see section below on selecting the appropriate level at which to code components). Fine-grained measurement of individual components that make up each program (or lesson) is beneficial for creating opportunities to observe variation in component experiences; if the components are coded too coarsely, it is likely that the coded components across programs will look very similar because of the limited opportunity for the coarse components to differ. However, if components are coded too finely, they may not reasonably be expected to move outcomes on their own.

**Fig. 3:**
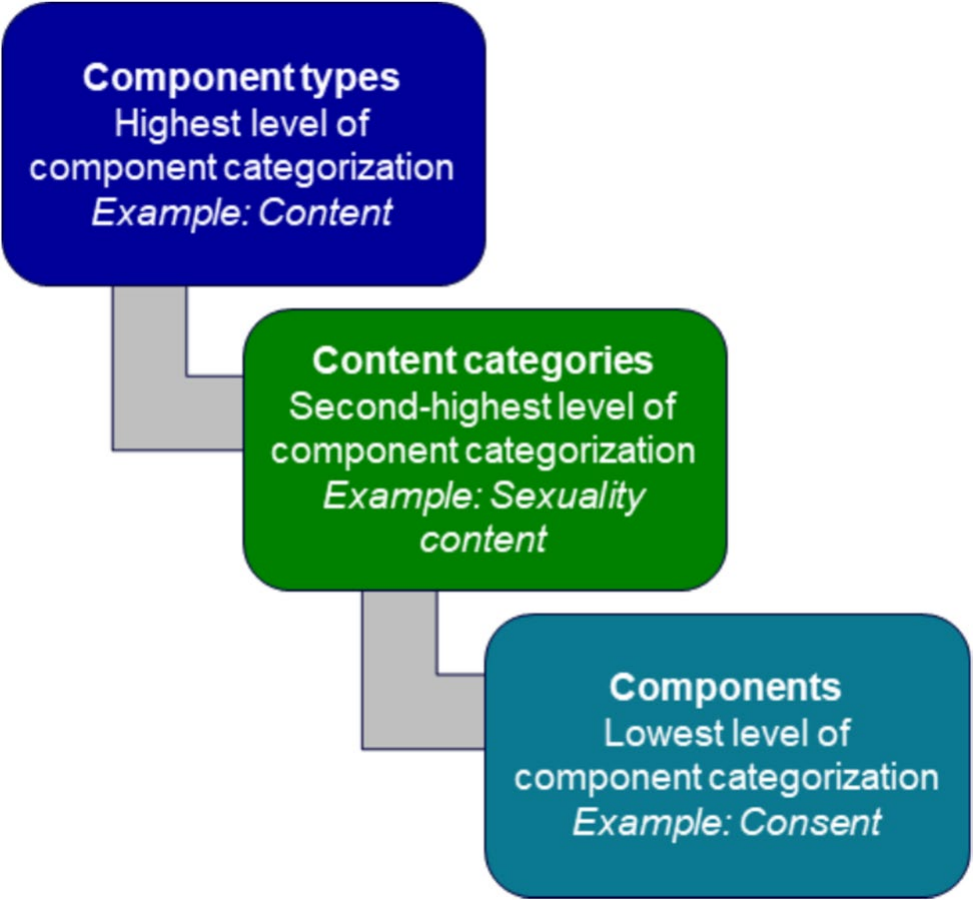
Hierarchy of granularity of components

A fine-grained checklist provides the option to aggregate categories at a later point. For example, each lesson or program session can be coded to indicate whether it contains content on alcohol substance use, other drug substance use, abstaining from substances, and ceasing use of substances. These components fall under the same component category, substance use content, and can later be collapsed at this higher, coarser level. Figure 3 (adapted from Knab et al. 2025) shows a hierarchy of how fine-grained content components measured in the tool were organized into content categories, which all fell under a broader content component type. Relatedly, it may be the case that some components (for instance, specific content and activities) are frequently offered in conjunction with each other within a given structural element (a lesson, in this study). In such situations, it may not make sense to develop and plan to use a tool that measures these components separately from each other. Instead, the tool might collapse them into a single unit (with a label that acknowledges features that span the individual components within a category).

For each individual component included in the checklist, there are response options for the user to indicate whether the component is part of a program or not, and whether the component is deemed to be “core” to the program. The instructions accompanying the checklist explain that core is a designation that program developers or distributors might make if they have a hypothesis based on existing theories or frameworks, or based on evidence from research that the components influence program outcomes.

#### Coding Components of the REA Program

After developing the tool for coding components, we selected the approach to coding REA’s program components. That is, we decided which level of the program to code, when to code them, and how to reliably code them.

##### Selecting the Appropriate Level at Which to Code Components

In order to maximize our opportunity to observe variation in component experiences, we used the most fine-grained approach available to us that available documentation would support: coding individual lessons based on information in the program manual. In doing so, we effectively treated each lesson as the structural element of interest, and disaggregated it into its component types (note, we did not document staffing, environment, or intended population component types, as these did not vary across lessons). This level of coding was feasible because there was sufficient information about the content and activities that were occurring within each of the 87 REA lessons in the program manual. Note: one could have coded the components of the REA program as a whole (rather than lesson-by-lesson); however, this would not have provided useful information about unique component experiences for individual youth to then use as predictors of variation in outcomes, which was the ultimate goal of this study.

##### Prospective Versus Retrospective Coding

Component coding can be done prospectively by coding program documentation to capture component experiences that are planned to occur, or it can be done retrospectively by observing or documenting the actual experiences of program participants during implementation. Below we discuss our decisions and approaches to prospective versus retrospective coding and our coding procedures in the Components Study of REA.

In an ideal situation under prospective coding, limited variation in component experiences will be observed, relative to what was expected when components were prospectively coded. This may occur when a highly structured/scripted program is being studied. Adaptations commonly occur, however, in standardized and non-standardized programs, and some interventions allow or encourage flexible implementation (Escoffery et al., 2018). In these situations, the components ultimately experienced may vary substantially from what was originally prospectively coded. Even in situations in which tools have been designed to appropriately measure flexible experiences (for instance, having facilitators report on adaptations), it is possible that unplanned adaptations may lead to unreliable measurement of actual component experiences under a prospective coding approach if reporting is unreliable or absent (Bishop et al., 2014).

Retrospective components coding, alternatively, can rely on observations or documentation of the actual experiences of program participants as their source material rather than a program manual. Therefore, retrospective coding may more reliably and accurately capture the component experience for program participants. However, retrospective coding is potentially resource intensive—all implementation experiences will need to be examined to carefully document the actual component experiences among study participants.

In the Components Study of REA, we conducted a prospective coding approach by using information about component experiences that were planned to occur. This was the most efficient approach for coding component experiences, and the only viable approach within the study budget. We first prospectively coded the components of all 87 REA lessons using the TPP program component checklist/taxonomy. We then created unique component profiles of each student through the combination of (1) this list of components associated with each of the 87 lessons, and (2) student attendance records (which were associated with the REA lessons offered on each day of attendance). Using these two sources of data, we were able to create counts of every component that each student uniquely experienced based on their attendance profile (See Knab et al., 2025 and Cole et al., 2025a, 2025b for details).

This was a much more efficient way of coding actual component experiences than attempting to retrospectively code every single instance of program implementation that occurred in each lesson (though, it likely sacrificed some precision when facilitators did not complete all activities as expected in a given lesson). In the Components Study of REA, facilitators reported that in 90 percent of lessons, all activities were fully completed (Asheer & Blesson, 2025). Given this high level of adherence, it is likely that the prospective coding produced an accurate measurement of component experiences.

##### Coding Procedures

To prepare for components coding for the Components Study of REA, we conducted a pilot test of the instrument. Two researchers on the project independently coded two selected REA lessons using the component checklist. We then compared their coding of these lessons and calculated a concordance statistic to assess inter-rater reliability. This pilot assessment confirmed that the tool was useful for the coding exercise, due to the high levels of inter-reliability rating (88% of the initial component codes for each lesson were in alignment; Cole et al., 2025a, 2025b). After piloting the coding tool and seeing that it showed promise, we trained staff to use the tool. Two independent coders reviewed each lesson in the curriculum and identified the specific content to be covered (e.g., content on goal setting, STI risk, drug use, or decision making), the activities used to deliver the content (e.g., discussions, games, role-playing or completing a handout), and the expected dosage. A third coder reconciled any differences between the two original coders.

### Linking Program Components to Outcomes

The goal of most component studies is to understand which components are responsible for driving outcomes. The identification of the “core components,” the subset of components that produce the desired outcomes among program participants, can be addressed empirically in a component study by measuring outcomes and exploring them as the result of variation in component experiences.

#### Selecting and Measuring Outcomes

Another important measurement consideration for component studies is appropriately identifying and measuring outcomes that might be affected by variation in component experiences (rather than exposure to the whole program). When studying program components, it is necessary to think about outcomes that are sufficiently sensitive to showing changes resulting from variation in component experiences. Often, outcomes appropriate for component research are proximal, more immediate than the policy relevant outcomes we typically examine in program evaluation research. That is, if we think about the logic model or theory of change for a program, we often consider the most policy relevant outcomes on the rightmost side of the logic model as the ultimate goal of the program; component research may need to explore antecedents to these policy-relevant outcomes. To identify these outcomes, it may be necessary to create component-specific logic models that link components of interest (e.g., different types of content or different structural elements) to measurable proximal outcomes to begin identifying potentially appropriate outcome measures (Cole & Murphy, 2016).

In the Components Study of REA, we developed and measured proximal outcomes that are tightly aligned with components. We examined all the lessons that made up the REA program and determined all the outcome constructs that were hypothesized to be affected by each of the lessons, in coordination with the program developer. We then identified survey items and scales to measure these outcome constructs. To vet the completeness and appropriateness of this outcome measurement approach, we convened a panel of individuals with expertise spanning components research, measurement, and outcomes likely to be observed from healthy relationship and TPP programs. During and after the meeting, the panelists provided feedback on the planned outcome constructs and approaches to measure them. Based on the panel’s feedback, we used the refined collection of survey items and scales to measure proximal outcomes likely to be sufficiently sensitive to variation in exposure to individual lessons.

#### Identifying Variation in Component Experiences to Predict Outcomes

In an impact evaluation (e.g., a randomized controlled trial), individuals may be assigned to a treatment program or a control condition, and, by comparing their outcomes, we can estimate the effect of the given program. In a component study, it is necessary to identify an analogous difference in program experiences across sample participants. In a prospective component study, it is possible to assign individuals or groups to conditions made up of specific component combinations to test the hypothesized effects of specific components on specific outcomes. However, the Components Study of REA was an exploratory study, and thus we did not have hypotheses about which of many possible components might be the drivers of outcome changes (Cole & Choi, 2020). Our study goal was to identify potentially promising components that could warrant a more rigorous evaluation of components (using a manipulated design) in the future. Thus, we capitalized on naturally occurring variation in component exposure across participants as the key predictors of outcome variance.

##### Leveraging Naturally Occurring Variation in Component Exposure

In the Components Study of REA, we had two sources of naturally occurring variation in component experiences to draw on: (1) Variation in lessons across S&Ss and (2) variation in youth attendance within S&Ss.

1. **Variation in Lessons Across S&Ss**: As described previously, the REA curriculum developer (the Center for Relationship Education) or its lead facilitators assembled an S&S made up of different REA curriculum lessons based on the school’s priorities and time constraints. As a result, naturally occurring variation in school priorities and constraints yielded variation in S&Ss and associated components. Ultimately, 27 schools implemented 40 different S&Ss in the Components Study of REA.
2. **Variation in Youth Attendance Within S&Ss**: Youth varied in terms of their attendance of lessons, which gave us an additional opportunity to capture variation in exposure to specific lessons (even among youth exposed to the same S&S). When youth were absent on a given day of an S&S, they were not exposed to the components of that day’s lessons. As described in the Prospective versus Retrospective coding section, our approach was to combine (1) the 87 prospectively coded lessons, and (2) individual student attendance records that showed which lessons they were exposed to, to generate a complete representation of all of the components that they ultimately experienced.

These two sources of variation allowed us to observe variation in lesson (and associated component) experiences by program participant, such that we could answer questions about whether youth who received more of an individual REA content component had better outcomes than youth who received less of that content component, for example.

#### Component Analysis

The goal in prospective component studies is to credibly link variation in component experiences to outcomes. Analytic approaches for component studies should follow the same best practices as other statistical analyses that explore relationships between predictors and outcomes—notably, approaches to (1) limit the role of omitted variable bias, (2) to protect against spurious, Type I errors, and (3) to lift up a useful and interpretable set of findings from exploratory analyses. Thus, in the Components Study of REA, we attempted to mitigate the omitted variable bias threat by adjusting for variables associated with component take-up and outcomes (Anastasio et al., 2025; Cole et al., 2025a, 2025b). To limit the threat of Type I errors, we limited interpretation to the set of statistically significant findings that are greater than the number expected to be observed from random sampling error (i.e., a version of the Bonferroni, 1936 correction).

There were two important analytic features to this component study that went beyond standard exploratory research approaches and helped to achieve the third goal of generating a useful and interpretable set of findings. First, we were flexible in how we operationalized component exposure. We originally expected to compare those individuals who were exposed to components with those who were not exposed to them. However, in practice, we saw that the key differentiator was the amount of component exposure, rather than a yes/no dichotomization of whether participants had any exposure to a given component; our analytic approach therefore took this into account (Knab et al., 2025). Second, the approach to reduce Type I errors was further enhanced by collapsing a large number of potential component predictors into a smaller number of reliable component categories; an additional benefit of this approach was that it yielded a more parsimonious and interpretable set of findings.

##### Capturing Variable Exposure to Individual Components

As noted above, we created a count of the number of times each individual experienced each type of content, activity, format (and other) components, by summing the components an individual experienced in each lesson (from the coded components data) across all lessons that person experienced (from the data showing variation in lesson attendance). As a result, our data set was not simply a collection of yes/no exposure measures of individual component experiences. Instead, we were able to measure a count of the number of times that each participant experienced each component in the TPP checklist across all lessons that they ultimately experienced. Articulating the amount of exposure to each component people ultimately experienced, rather than a dichotomized indicator of whether a component was experienced, ended up being an important detail in our analysis: Knab et al. (2025) pointed out that the dichotomized version of component experience masked substantial variability in the actual component experiences of youth. In this exploratory component analysis, we found that a flexible and data-driven approach was a more appropriate analytic strategy than strictly pursuing our original analytic plans.

##### Pooling Individual Component Variables into Component Categories

As noted above, we conducted a fine-grained coding of program components of REA and aimed to assess these variables as predictors of outcomes. In our initial exploration of components as predictors of outcomes, there was a great deal of noise in the findings when we examined the many fine-grained components as separate predictors of outcomes. That is, our initial analyses had no clear pattern of direction or statistical significance of associations. As a result, in our ultimate data preparation and analysis, we pooled fine-grained components from the same substantive category together into a component category (as shown in Fig. 3). This higher-order organization of individual components helped us use a more reliable, informative, and parsimonious collection of REA program components in our analysis. This smaller collection of predictor variables in the exploratory analysis reduced the number of potential Type I errors, while simultaneously yielding a simpler story about the components that were most predictive of outcomes.

## Discussion

Component studies and analyses can provide audiences with useful information about the ingredients of programs that drive outcomes: the core components of the programs (Blase & Fixsen, 2013; Forrester & Cole, 2023b). Evidence supporting core components can help program developers create curricula, decide how to adapt programs, or enhance existing programs as part of continuous quality improvement. These core components are the elements that will need to be implemented well to achieve intended outcomes (Ferber et al., 2019). In addition, in some instances, core components can be implemented as standalone or supplements to programs to address programmatic needs and service gaps (Asheer & Aharpour, 2025). Providing effective components to populations might be a way to address disparities in outcomes and reduce service inequities, when full programs are too expensive or infeasible to implement in a given setting (Butler, 2020; Castillo-Sumi, 2020).

The need for this article, a summary of practical considerations in components study research, emerged from our own Components Study of REAL Essentials. While a number of instructional publications exist to offer guidance to researchers conducting exploratory studies of components of programs, we found that the process of documenting, measuring, and identifying core components was particularly complex and multi-faceted in practice. Given that extant literature guiding components research practice did not offer sufficient guidance for this exploratory study, an article describing our successes and challenges would be a contribution to the field and would help future researchers conducting exploratory component studies in this burgeoning area of research. Below, we provide recommendations for researchers designing and executing component studies based on the successes, missteps, and best practices that emerged out of our exploratory examination of components. We present these recommendations in general terms to be useful guide points for prevention researchers at large, rather than specific to the TPP field.

### 1) Establish an Interpretable Unit of Component Experience

In the Components Study of REA, youth varied from each other in the lessons that they experienced (due to variation in S&Ss and individual attendance). However, most prevention audiences do not have sufficient knowledge about the individual lessons of the REA program for this to be a meaningful unit of experience for reporting. Audiences would not be familiar enough with the content or activities of any given lesson to understand whether or why the lesson was associated with outcomes. To produce a more useful and interpretable set of findings, we recoded each lesson into its component types and used component types as the key predictor variables in our analysis, to tell a story about program content, activities, and dosage. This approach of transforming non-relevant structural elements into more useful component types is an important practice that can be used by future researchers conducting components studies of prevention programs. LoBraico et al. (2019) offers an example of such a study; these authors decomposed the Strengthening Families Program: for Parents and Youth Ages 10–14 into six broad, well-defined types of content (e.g., parental monitoring and behavior management, positive family relationships, etc.), and used these as meaningful, interpretable units of component experience that varied across their study sample.

### 2) Conduct Precise and Theory-Informed Outcome Measurement

In the Components Study of REA, we (1) identified outcome constructs that would be sensitive to variation in component experiences (which are narrower in scope than a full program), (2) worked with an expert panel to vet and refine those measures, and (3) administered a survey that enabled these constructs to be carefully measured at multiple timepoints. Appendix A of Cole et al., (2025a, 2025b) contains the individual items and constructs that were developed from this process, which illustrate the kinds of precise, theory-informed outcomes that can be generated from such a process.

Our approach to developing an instrument sufficiently sensitive to capture outcome changes based on differences in small-component experiences was successful. This approach to developing an outcome measurement that is closely aligned with the components of a curriculum can be used in prospective component research studies of prevention programs and can be conceptualized as an application of the IM approach described earlier (Fernandez et al., 2019).

### 3) Aggregate Component Experiences to Capture Substantively Meaningful Differences

Using the TPP component checklist as a taxonomy against which to code components enabled more interpretable units that showed variation in components than if we focused on higher-level structural elements. However, the checklist also introduced subtle differences in component experiences across individuals whose practical component exposure was substantively the same. Thus, there were too many different component experiences to explore as drivers of outcomes, and many of the differences in component experiences were much too subtle to be expected to influence outcomes by themselves. By aggregating minor differences in component experiences into broader categories, we were able to generate more parsimonious and more reliable metrics of how individuals varied in their component experiences. As shown in Knab et al. (2025), variation in fine-grained content experiences around alcohol and drug use, brain development, cessation and abstinence around substance use could all be conceptualized as variation in experiences of a coarser “Physical health – substance use” content category, and Anastasio et al. (2025) found that this component category was a strong, significant predictor of several outcomes (conflict management, self-awareness, social support, future orientation, and responsible decision-making). Cole et al. (2025a) found in a meta-regression that evidence-based TPP programs with a higher count of components in this category tended to have larger impacts. Component research studies that use a taxonomy to conduct fine-grained coding of components can use a similar aggregation approach to coarsen observed components into reliable categories that will enhance the interpretability of their findings.

### 4) Ensure that the Coding of Components from a Taxonomy Creates Useful and Interpretable Units of Interpretation

The TPP checklist allows programs (or their structural elements, like the lessons of REA) to be separately coded in terms of content, activity, format, etc. However, a limitation of this coding approach as applied in the Components Study is that each of these component types was coded independently. We coded each lesson in terms of the content, activities, formats that were found in the lesson (i.e. the various component types found in the checklist); however, the coding did not capture the combinations of these types—that is, while we knew that content components A, B, and C were found in a given lesson, using activities M, and N, and using formats X, and Y, we didn’t know whether content A was taught using activity M or N, or if it was done using format X or Y. And as a result, the eventual analysis that linked components to outcomes (found in Anastasio et al., 2025) did not analyze combinations of components as drivers of change in outcomes. Therefore, we were unable to know whether the finding about a potentially promising type of content was solely attributable to content alone, rather than the activity or mode in which that content was delivered. Future component researchers may address this problem by using a taxonomy of components that more fully represents combinations of component types; this might also more easily be accomplished by focusing on well-defined and understood structural elements, rather than individual components, as the components of interest.

Alternatively, Coyle et al. (2013) used a randomized design to test the relative effectiveness of a community service-learning structural element as a well-defined unit of component experience. This service-learning structural element can be conceptualized as a combination of content (information provided during the experience), environment (an outside, non-traditional setting), delivery mechanism (volunteer service), and other component types. Focusing on the structural element of the service-learning experience as the unit of inference (rather than any of these individual types) allows for interpretation of the impact of this broad experience of the structural element—a practical point of interpretation—rather than any of the individual component types that make up the service-learning experience.

### 5) Anticipate Difficulties Identifying Signals of Effectiveness from Noise

In our exploratory component study with many candidate components and a large number of proximal outcomes, several component-by-outcome combinations were examined (even after we grouped individual components into categories). In our analysis, we observed several statistically significant findings that linked components to outcomes that ran counter to expectation, and concluded that many of them may have been Type I errors due to the multiple hypothesis testing problem. Future research examining components of prevention programs can potentially better address the multiplicity problem by (1) paring back the number of components of interest in the taxonomy, and (2) potentially limiting the number of outcomes of interest to a subset of the most policy-relevant outcomes (even if this means sacrificing some less-relevant outcomes that are likely to be sensitive to showing differences resulting from variation in component experience). Prespecifying hypotheses about the most promising subset of component and outcome combinations (and, ideally, registering this information externally to guard against any concerns about *p*-hacking) would be an appropriate way for future researchers to better address this issue (Gelman & Loken, 2014; Wasserstein & Lazar, 2016).

### 6) Consider Approaches to Identify “What (Components) Work for Whom”

The Components Study of REA was implemented shortly after the end of the COVID-19 lockdown period, where recruitment of schools into a research study was a substantial challenge. As a result, the study suffered lower-than-expected power to detect the relationship between narrowly defined components and outcomes using the full study sample. Thus, we did not attempt to further explore the heterogeneity of the relationships between components and outcomes across subgroups of the study sample. Future researchers conducting components studies may want to explore the differential effectiveness of program components and plan to recruit large enough samples to be well powered to detect these relationships more credibly. In addition, researchers can make their data available to others to enable these types of explorations to occur in the future using larger, pooled datasets (Grant et al., 2022). Researchers who are interested in exploring this opportunity using the Components Study of REA data can access them at the Inter-university Consortium for Political and Social Research (Knab & Cole, 2025).

## Conclusion

As policymakers are faced with competing demands and ever-smaller budgets, they need explicit guidance about opportunities for maximally effective programming for specific populations or communities. To make evidence-informed decisions about which program components are worth implementing, when, for whom, and in what contexts, researchers must conduct more and better research on components that yield consensus about which components are most important for program effectiveness. We hope that the guidance provided in this article can serve as a helpful foundation for researchers within and outside of the TPP field to continue building the evidence for components.

## Supplementary Information

Below is the link to the electronic supplementary material.

ESM 1 (PDF 366 KB)

## Data Availability

The data from the Components Study of REAL Essentials are available for download at ICPSR at https://www.icpsr.umich.edu/web/ICPSR/studies/39494, reference number ICPSR 39494.

## Declarations

### Clinical Trial Registry

The Components Study of REAL Essentials was registered at clinicaltrials.gov (NCT06112912, protocol ID 75P00120F37051) on 10/30/2023.
