# Supplementary material for: Strategies for Identifying Core Components of Programs: an Exploratory Descriptive Component Case Study of a Teen Pregnancy Prevention Program
Source: Prev Sci. 2026 Feb 10;27(2):279–92. doi: 10.1007/s11121-026-01881-8 (PMC12999639; doi:10.1007/s11121-026-01881-8)
Supplement: Supplementary file 1 — (PDF 366 KB) [file 11121_2026_1881_MOESM1_ESM.pdf]

Checklist

Component	Definition	Component present?	Is this component core?	Core component justification	Lesson number(s)/ activities where present (e.g., Lesson 4, manual page 95) *Provide only one location for each component*	Notes (e.g., describe what is in "other")	Allowable and unallowable adaptions (optional)
**Content: The intended subject matter being provided**							
Anatomy/physiology	Content about the structure and function of sexual and reproductive organs						
Contraception - Condoms	Content about condoms worn on the penis or inserted into the vagina to prevent pregnancy and STI transmissions						
Contraception - Long-acting reversible contraceptives	Content about long-acting, reversible contraceptives (LARCs), including implants and intrauterine devices (IUDs)						
Contraception - Other	Content about other types of contraception or birth control methods, including but not limited to diaphragms, sponges, spermicide, emergency contraception, and withdrawal						
Contraception - Pills, patches, rings, and shots	Content about methods of contraception that are used on a schedule, including injections, pills, vaginal rings, and skin patches						
Maternal health	Content about the health of women during pregnancy, childbirth, and the postnatal period						
Puberty/development	Content about the physical, psychological, and emotional changes during the progression from childhood to adulthood						
Reproduction	Content about pregnancy, including how an egg is fertilized, the development of an embryo and fetus, and maternal changes						
Risk of STIs and Pregnancy	Content aiming to increase students' awareness that pregnancy and STI infections could happen to them						
Sexual health	Content about the state of physical, emotional, mental, and social well-being in relation to all aspects of sexuality and reproduction. Sexual health includes a positive and respectful approach to sexuality and sexual relationships, as well as the possibility of having pleasurable and safe sexual experiences, free of coercion, discrimination, and violence.						
Sexual orientation	Content about the people an individual is sexually attracted to. Sexual orientation is an enduring pattern of romantic or sexual attraction to persons of the opposite sex, the same sex, or to both sexes.						
Sexual risk avoidance	Content covering abstinence and avoidance of sexual behaviors (including choosing not to have sex) to minimize risk of adverse outcomes						
Sexual risk discontinuation	Content offered to youth who have previously had sex to promote discontinuation of sexual behaviors and thereby avoid STIs and unintended pregnancy						
Sexual risk reduction	Content to promote reduction of sexual behaviors to improve protection against STIs and unwanted teen pregnancy. The content often includes messages about abstinence as well as information about condoms and contraception						
STIs - Information	General information related to sexually transmitted infections caused by bacteria, viruses, or parasites						
STIs - Prevention	Content about prevention of STIs, including topics on abstinence and condoms and other protective barriers						
STIs - Screening	Content about health testing for STIs, including information on accessing locally available testing sites						
STIs - Treatment	Content about treatment/measures intended to cure or reduce symptoms of STIs, often using antibiotics						
STIs - Vaccination	Content covering inoculation to ensure active acquired immunity or protection from certain STIs (including PrEP for HIV)						
Body image	Content related to the mental representation a young person has of their physical self at any given point in time. Body image can either positively or negatively impact a young person's ability to make healthy decisions related to sex and relationships						
Consent	Content about the process of having partners be fully informed and agreeing to sexual activity; role of how alcohol and drug use can affect consent						
Decision making	Content related to how youth make decisions about sex and relationships. Content may also include activities for youth to build decision making skills						
Healthy relationships	Content about what constitutes a healthy versus unhealthy relationship, including relationship red flags						
Healthy romantic relationships	Content about romantic relationships that reinforce physical, psychological, and emotional well-being. Includes characteristics of healthy and abusive romantic relationships						
Sexual health needs and experiences of LGB youth	Content about the sexual health needs and experiences of lesbian, gay, and bisexual individuals						
Social media, texting and online interactions	Content on how media, including social media and texting, and exposure to Internet pornography can influence youths' ideas about sex and sexuality						
Values and sexuality	Content on how values may influence a young person's thoughts, feelings, and behaviors related to sexuality						
Brain development & emotions	Content about the changes the brain undergoes and the impacts these changes have on youth feelings and emotions						
Cognitive behavioral therapy (CBT)	Content about the psycho-social intervention intended to improve mental health by identifying negative thoughts and replacing them with more objective, positive, and realistic thoughts, with the ultimate goal of improving behavior						
Empathy	Content about understanding and respecting the views and feelings of others						
Goal setting	Content about the development of an action plan designed to motivate oneself toward a goal						
Meditation	Content about training attention and awareness with the goal of achieving a mentally clear and emotionally stable state						

Component	Definition	Component present?	Is this component core?	Core component justification	Lesson number(s)/ activities where present (e.g., Lesson 4, manual page 95) *Provide only one location for each component*	Notes (e.g., describe what is in "other")	Allowable and unallowable adaptions (optional)
Mindfulness	Content about achieving and maintaining a mental state of full awareness of the present moment, acknowledging one's feelings, thoughts, physical sensations, and surrounding environment.						
Motivational interviewing	Content about the person-centered form of counseling to encourage motivation for change						
Personal vulnerability	Content about putting oneself out there, living authentically, and sharing one's feelings and experiences with those they trust (such as with a partner)						
Resilience	Content about letting go of stress and accessing and sustaining positive emotion						
Self-efficacy/empowerment	Content about an individual's belief and confidence in their ability to execute behaviors aligning with their own motivation, character, and social environment						
Self-esteem	Content about an individual's perception and general opinion about themselves, essential to good mental health						
Self-regulation	Content about skills for managing one's emotions, stress, and behaviors						
Alternative schooling	Content on educational establishments that have nontraditional curriculum and methods						
College preparation	Content about preparatory measures for admission and attendance at a college/university, college bridge programs, financial aid, support for foster youth, and support for first generation college students						
Graduating from high school	Content about graduating with a high school diploma or GED						
School engagement	Content about how students engage with aspects of learning and education						
Supplemental academic services	Content about additional academic help beyond what is typically offered in schools, such as tutorial or remedial assistance in certain subjects, etc.						
Vocational/skills training	Content about education that prepares a person to take up employment in a skilled craft or trade, such as career and technical education (CTE) classes						
Brain development & substance use	Content about how adaptation and development of the brain can be negatively impacted by heavy substance use						
Substance use - Abstinence	Content about avoidance of and non-engagement in potentially addictive substances or behavior						
Substance use - Alcohol	Content about beer, wine, and spirits that depress the central nervous system in high doses, and how it affects decision making						
Substance use - Other drugs	Content about other products that can be smoked, ingested, injected, or inhaled to produce a physiological change that may affect decision making or increase risk of disease						
Substance use cessation	Content about discontinuing use of a potentially addictive substance in the long term						
Boundary setting/refusal skills	Content about ways to identify boundaries for acceptable behavior, and learning how to refuse when being pressured to do something outside of those boundaries						
Child development	Content about the sequence of physical, language, thought, and emotional changes that occur within a child from birth through adulthood						
Communication skills	Content about verbal and nonverbal skills and cues used for social interaction and cognition						
Conflict resolution/social problem solving	Content about disagreement and conflict, and identifying peaceful and mutually acceptable resolutions to that conflict						
Connections with trusted adults	Content about the benefits of having a positive relationship with a trusted adult, including a parent(s)						
Cultural values	Content about the core customs, ideologies, and principles shared by a specific culture or group of people in a community						
Sense of self	Content about an individual's personal perception of themselves						
Biological sex roles	Content about the range of behaviors and attitudes considered appropriate and acceptable for a person based on their biological sex						
Leadership	Content about being a leader, motivating others to achieve a common goal						
Normative beliefs	Content about the beliefs and behaviors that society agrees to be appropriate and/or correct						
Parenting skills	Content about the skills that parents need in order to fulfill their duties as an effective and loving parent						
Social competence	Content about the social skills necessary to handle social interactions effectively						
Social influence/actual vs. perceived social norms	Content about what society or a community regards as typical or standard behavior (e.g., social norms) and how these norms affect individual decision making and behavior						
Social support/capital	Content about the value of social relationships as a source of support and information to individuals						
Self-discovery	Content about how an individual develops a clear and unique view of themselves						
Morals/values	Content about standards of behavior, principles, or beliefs that are used to judge what is acceptable to do (i.e., right or wrong)						
Spirituality	Content about a connection with something greater than oneself						
Volunteering/civic engagement	Content about individual and collective actions intended to address a public issue/concern						
Other	Other types of content not captured in the list of content components as one of the components described above (*describe in notes field*)						
Other	Other types of content not captured in the list of content components as one of the components described above (*describe in notes field*)						
Other	Other types of content not captured in the list of content components as one of the components described above (*describe in notes field*)						

Component	Definition	Component present?	Is this component core?	Core component justification	Lesson number(s)/ activities where present (e.g., Lesson 4, manual page 95) *Provide only one location for each component*	Notes (e.g., describe what is in "other")	Allowable and unallowable adaptions (optional)
**Delivery mechanism: The intended principles and practices by which the content is provided**							
Method: Anonymous question box	Method includes an anonymized source for questions from students within program						
Method: Artistic expression	Method includes creating music, dancing, sculpting, drawing, painting etc.						
Method: Assessment/survey	Method includes assessments or surveys intended to measure an individual's knowledge or ability to complete a particular task						
Method: Booster session	Method includes additional sessions traditionally offered after the completion of the program, to reinforce material previously offered						
Method: Case management	Method of assessing, planning, monitoring, and evaluating services provided to a child						
Method: Cognitive behavioral therapy (CBT)	Method of delivering content intended to improve mental health by identifying negative thoughts with participants, and replacing them with more objective, positive, and realistic thoughts						
Method: Demonstration	Method includes using examples or experiments to explain things or make them clear						
Method: Discussion/debrief	Method includes a conversation or debate intended to explore or review a topic						
Method: Family session	Method includes a session with family members						
Method: Game	Method includes playing a game or sport						
Method: Home visiting	Method of supporting pregnant and parenting families, accomplished by having implementers travel to a participant's home for service delivery						
Method: Homework assignment	Method includes assignments completed outside of the implementation setting						
Method: In-session assignment	Method includes assignments completed in the implementation setting						
Method: Introduction	Method includes an introduction activities to establish group norms, goals for the program, icebreaker activities, etc.						
Method: Lecture	Method includes discourse given to an audience or class						
Method: Motivational interviewing	Method includes use of person-centered counseling to encourage motivation for change						
Method: Music	Method includes listening to music						
Method: Parent-focused activity	Method includes activity or assignment incorporating parental involvement and/or input						
Method: Peer-to-peer	Method includes peers delivering program materials to their peers.						
Method: Public service announcement	Method includes a publicly disseminated message to raise awareness of and influence attitudes on a social issue						
Method: Reading	Method includes participants reading program material						
Method: Role play/Practice	Method includes participants assuming and acting out roles based on various situations, or practicing skills in various situations						
Method: Self-guided activity	Method includes activity conducted by the youth for themselves without additional input/interaction						
Method: Service learning	Method includes experiential education, often conducted as volunteer activity outside of the standard implementation setting						
Method: Slide show	Method includes presentation of projected images and/or photographic slides						
Method: Social media	Method includes delivery of material through social media (e.g., Instagram, TikTok)						
Method: Spiral learning	Method of returning to content multiple times, based on premise that a student learns more about a subject each time the topic is encountered						
Method: Storytelling	Method includes an activity of telling or writing stories						
Method: Text message	Method includes text messages sent to youth's or caregiver's cellular phone						
Method: Video	Method includes recording of moving visual images, often accompanied by audio						
Method: Other	Other types of delivery mechanism not captured above (*describe in notes field*)						
Method: Other	Other types of delivery mechanism not captured above (*describe in notes field*)						
Method: Other	Other types of delivery mechanism not captured above (*describe in notes field*)						
**Dosage: The intended duration, frequency, and intensity of the program**							
Frequency	How often the program occurs in a given period (e.g. once a day for 3 weeks, for a total of 15 lessons (*describe in notes field)*						
Intensity	The length of time for a typical intervention encounter/lesson/session (e.g. each lesson occurs during a 40 minute classroom period) (*describe in notes field)*						
Duration	The total period of time over which the intervention occurs (e.g. the intervention includes a total of 10 hours [600 minutes] of programming) (*describe in notes field)*						
Other	Other types of dosage not captured above (*describe in notes field)*						
**Staffing: The intended training and characteristics of the individual(s) delivering the content**							
Deliverer: Community health worker	Program is delivered by a community member employed by an organization or community						
Deliverer: Faith-based individual	Program is delivered by an individual affiliated with a specific religion or religious group						
Deliverer: Health educator	Program is delivered by a professional who educates people on matters of health						
Deliverer: Mental health provider	Program is delivered by a professional who provides treatment for mental health diagnoses						
Deliverer: Mentors	Program is delivered by an individual who provides guidance, motivation, emotional support, and role modeling						

Component	Definition	Component present?	Is this component core?	Core component justification	Lesson number(s)/ activities where present (e.g., Lesson 4, manual page 95) *Provide only one location for each component*	Notes (e.g., describe what is in "other")	Allowable and unallowable adaptions (optional)
Deliverer: Parents/family	Program is delivered by caregivers and legal guardians of an individual.						
Deliverer: Peers	Program is delivered by a group of people with interests, background, age, and/or social status similar to those of the intended population						
Deliverer: Primary care provider	Program is delivered by a provider of day-to-day health care (for example, doctors, nurses, medical assistants) responsible for the ongoing health of their patients						
Deliverer: Social worker and/or counselor	Program is delivered by a professional focused on promoting social change, development, and empowerment of people and communities						
Deliverer: Teachers	Program is delivered by a person who provides instruction to students, and is employed by a school (district)						
Deliverer: Other	Other kinds of individuals providing services (*provide title and other details in notes field*)						
Experience: Credentials	Certifications or other credentials required of implementing staff (*describe in notes field*)						
Experience: Education	Educational experience, background, expected of program implementer (*describe in notes field*)						
Experience: Other	Other experience expected of individuals providing services						
Number of staff providing services	Indicate the number of individuals providing services to a given set of youth in the notes field. In many cases, this will be 1. When co-facilitation occurs, this number will be greater than 1 (*please describe in the notes field*)						
Training: Additional training required	Program implementer is required to receive additional training other than developer-led training (*describe in notes field, including duration*)						
Training: Developer-led training required	Program implementer is required to receive training offered and supported by program developers (*indicate duration in notes field*)						
Training: Other	Other training or TA provided to support program delivery (*describe in notes field*)						
Other	Other types of staffing not captured above (*describe in notes field*)						
Other	Other types of staffing not captured above (*describe in notes field*)						
Other	Other types of staffing not captured above (*describe in notes field*)						
**Format: The intended structure and organization by which program content is delivered**							
Group size: Full-group activity	Activities conducted with the entire target group						
Group size: Independent/individual activity	Activities conducted on an individual basis						
Group size: Small-group activity	Activities conducted with small groups of youth (for example, four or fewer)						
Group size: Other	Activities conducted in an alternate group size (*describe in notes field)*						
Mode: In-person	Participants are physically present						
Mode: Phone (audio)	Participants engage over the phone for programming delivered as audio						
Mode: Phone (text)	Participants engage over the phone for programming delivered through text messages						
Mode: Phone (app)	Participants engage over the phone for programming delivered through an online application						
Mode: Online/computer (asynchronous)	Participants engage through self-directed online means (call, online lessons, etc.)						
Mode: Online/computer (synchronous)	Participants engage through online means (call, online lessons, etc.)						
Mode: Other	Other modes of engagement with programming (*describe in notes field)*						
Other	Other types of formats not captured above (*describe in notes field)*						
Other	Other types of formats not captured above (*describe in notes field)*						
Other	Other types of formats not captured above (*describe in notes field)*						
**Environment: The intended settings or locations where the program occurs**							
Context: Environmental supports	Physical and social supports within the implementation environment (e.g., school climate, community/civic/governmental support, academic environment, etc.) *(describe in notes field)*						
Context: Environmental constraints	Physical and social constraints within the implementation environment (e.g., school climate, community/civic/governmental support, academic environment, etc.) *(describe in notes field)*						
Context: Other	Other types of contextual supports and constraints (*describe in notes field)*						
Setting: After school	Programming is delivered to youth after the conclusion of the regular school day						
Setting: Community based	Programming is delivered to youth in a community setting						
Setting: Correctional facility	Programming is delivered to youth in a correctional facility						
Setting: Faith based	Programming is delivered to youth in a religious or faith-based setting						
Setting: Health care clinic	Programming is delivered to youth in a health care clinic						
Setting: Home-based case management	Programming is delivered to youth in their home during case management						
Setting: Home/housing	Programming is delivered to youth in their home						
Setting: In school (during the school day)	Programming is delivered in an institution that provides instruction to students within a particular discipline during regular school hours. If appropriate, please describe in the notes field the specific classroom/subject (for example, health class) where programming should occur						
Setting: Mental health clinic	Programming is delivered to youth in a mental health clinic						
Setting: Residential facility	Programming is delivered to youth in a residential facility						
Setting: School-based health clinic	Programming is delivered to youth in a school-based health center						
Setting: Other	Other type of setting where programming is provided (*describe in notes field)*						
Other	Other types of environments not captured above (*describe in notes field)*						
Other	Other types of environments not captured above (*describe in notes field)*						
Other	Other types of environments not captured above (*describe in notes field)*						
**Intended population characteristics: The characteristics of the intended population receiving the program**							

Component	Definition	Component present?	Is this component core?	Core component justification	Lesson number(s)/ activities where present (e.g., Lesson 4, manual page 95) *Provide only one location for each component*	Notes (e.g., describe what is in "other")	Allowable and unallowable adaptions (optional)
Age or grade in school	Age or grade in school of youth in intended population *(describe in notes field)*						
Biological sex	Sex of youth in the intended population *(describe in notes field)*						
Ethnicity	Ethnicities of youth in the intended population *(describe in notes field)*						
LGB	Majority of the intended population consider themselves lesbian, gay, or bisexual						
Race	Race(s) of the youth in the intended population *(describe in notes field)*						
Urbanicity	Urbanicity of the indented population *(select from drop down)*						
Homeless or runaway youth	Youth participants do not have safe or stable housing						
Youth with developmental disabilities	Youth participants have developmental disabilities						
Sexually active youth	Youth participants are sexually active						
Pregnant/expectant or parenting	Youth participants are pregnant/expectant or parenting						
Child welfare	Youth participants are involved in the child welfare system						
Juvenile justice	Youth participants are involved in the juvenile justice system						
Population-specific needs/challenges	Specific needs and challenges of the intended population that the program is intended to address *(describe in notes field)*						
Population-specific strengths	Specific strengths of the intended population that the program requires for implementation *(describe in notes field)*						
Other	Other types of intended population features not captured above (*describe in notes field)*						
Other	Other types of intended population features not captured above (*describe in notes field)*						
Other	Other types of intended population features not captured above (*describe in notes field)*						
